# Tackling health inequalities in a public health organization: the case of the Barcelona Public Health Agency

**DOI:** 10.1186/s12939-022-01724-2

**Published:** 2022-09-10

**Authors:** Gloria Pérez, M. Isabel Pasarín, Vanessa Puig-Barrachina, Katherine Pérez, Maica Rodríguez-Sanz, Lucia Artazcoz, Carme Borrell

**Affiliations:** 1 Agència de Salut Pública de Barcelona (ASPB), Plaça Lesseps 1, 08023 Barcelona, Spain; 2 Experimental and Health Sciences Department, Universitat Pompeu Fabra, Carrer Dr Aiguader 80, 08003 Barcelona, Spain; 3 Centro de Investigación Biomédica en Red de Epidemiología y Salut Pública (CIBERESP), C/ Monforte de Lemos 3-5. Pabellón 11. Planta 0, Madrid 28029 Madrid, Spain; 4 Institut d’Investigació Biomèdica Sant Pau (IIB SANT PAU), Carrer Sant Quintí, 77-79, 08041 Barcelona, Spain

**Keywords:** Inequalities, Public health, Participatory research, Policies

## Abstract

**Background:**

Municipalities are important actors in the implementation of policies to tackle health inequalities, which requires political will, the availability of financial support, and technical and human resources. With the aim of aligning with local government political priorities, in 2017 the Barcelona Public Health Agency (Agència de Salut Pública de Barcelona, henceforth ASPB), which is responsible for the public health functions of the city, launched a strategy to improve the approach to tackling health inequalities in all its services. The objectives of this study were to show how social health inequalities were addressed in the ASPB from 2017 to 19 and to describe which actions were proposed after a participatory process aiming to create a plan to systematically incorporate health inequalities in ASPB actions.

**Methods:**

The ASPB has 304 workers, 8 directors and 20 services or departments. Participatory methodologies were carried out: 1) semi-structured interviews with department heads (*N* = 12, 60%); 2) world cafe workshops open to a group of workers (*N* = 63, 37%); 3) a *Quick and Colorful* voting session open to a group of workers (*N* = 108, 63%); and 4) Hanlon matrix with 19 actions to be prioritized (*N* = 12 services, 60%).

**Results:**

Semi-structured interviews and world cafe workshops provided 40 potential actions. After a step by step process of participatory prioritization, seven lines of action emerged: 1) to make progress in collaborative networking; 2) to promote policy evaluation; 3) to increase the ability of the ASPB to evaluate policies to reduce health inequalities; 4) to incorporate the axes of inequalities in all ASPB products; 5) to improve information on vulnerable groups; 6) to incorporate the gender perspective; and 7) to participate in an internal training plan to address health inequalities.

**Conclusions:**

The participation of ASPB public health professionals and staff allowed the organization to design a shared plan of actions to address health inequalities. This experience could be useful for other municipalities whose political agendas include tackling inequalities in health.

## Introduction

Tackling health inequalities at the local level requires specific actions on the political agenda, the availability of financial support, and technical and human resources [1, 2]. Local strategies dealing with health inequalities are usually based on instructions from the local authority to the technical workforce to achieve sectorial objectives. However, a number of recent documents have reinforced the idea that people working in the public health sector play an important role in reducing health inequalities [3]. Furthermore, a disconnect between the local authority and employees can lead to a failure to achieve political objectives and proposed activities [4]. In addition, to have an impact on health equity, health professionals need to develop appropriate skills and attitudes to allow them to become advocates for change [5].

In the city of Barcelona, the arrival of a new left wing party in local government in 2015 was a substantial boost the role of reducing health inequalities in the political agenda. Two of these are “The strategy for inclusion and the reduction of inequalities” [6] and “The Neighbourhoods Plan” [7], which included the participation of the ASPB. In this context, the Barcelona Public Health Agency (Agència de Salut Pública de Barcelona, henceforth ASPB) had the opportunity to participate and lead most of the political spaces that opened up at that time.

The ASPB has demonstrated leadership in tackling health inequalities [8] describing them [9–12] and intervening to reduce them [13]. The ASPB is responsible for public health in the city (not health services) and, although it has attempted to decrease social inequalities, the intensity of activities depends not only on the political will of each government, but also to the intrinsic activity of each department and on the skills of the workforce. Taking advantage of the favorable political context after 2015, we decided to generate a plan to identify the potential contribution of public health workers to reducing inequalities in health in the city, as well as the worker’s shortfalls or needs in taking such action. The objectives of this study were to describe how social and health inequalities were addressed in the ASPB from 2017 to 19 and the actions proposed after a participatory process to design a plan to systematically incorporate health inequalities in ASPB actions.

## Methods

The ASPB has 304 workers in different areas of public health (including 1 managing director, 8 directors and 20 head of services or departments). In the first stage of the project, we selected all areas except for health protection and laboratory services because these are the departments that are least likely to work with health inequalities in their daily work. The workers included in the initial stage were 172 employees (56%).

The stages were:

1. The leading group was constituted by the managing director, 2 directors, 2 heads of service/department and 3 public health technical staff, all of whom are experts in health inequalities.
2. Diagnostic phase consisted first of semi-structured interviews with heads of departments or directorates (Table 1). Second, a world cafe workshop [14, 15] with all participating workers was held to answer 2 questions: i) how do you address the determinant of inequalities (e.g. housing, occupation etc.) in the service where you work? ii) What could be done to improve the performance of the service where you work in relation to this determinant? At the end of the session, a summary list was obtained of the points discussed at each table and session of the world cafe. Following the conceptual framework of social determinants of health in urban areas used in the ASPB [16] in this phase, 2 participatory sessions were proposed. In each of the sessions, there was a conversation table for each of the determinants of the conceptual model itself with a total of 10 tables: urban planning and mobility; housing, environment, food safety, occupation, education, social transfers, health and social services, participation and citizenship, and family settings. Each table consisted of 4 to 5 participants and one of the participants was the reporter, who had previously attended a training session to encourage discussion and to collect and present the conclusions of each table.

**Table 1:** Checklist for the semi-structured interview in the plan for addressing health inequalities in the Barcelona Public Health Agency

The purpose of this checklist is to promote internal reflection on the degree to which health inequalities are addressed during the formulation and implementation of the lines of work of the ASPB’s services portfolio and to identify the actions in which the ASPB has to incorporate health inequalities in order to improve its performance. The following checklist will apply to each of the lines of work described in the ASPB’s services portfolio. As the person in charge of the line of work, you have to answer the following questions, always with reference only to the line of work.							
Name of the service or directorate:							
Name and surname of the person or persons who filled out this checklist:							
**1. Objectives**							
Describe the main objectives of your service or directorate:							
**2. Population**							
						Yes	No
Are there any goals in your service or directorate aimed at a specific population group? (mark the answer)							
If the answer to the previous question is YES, describe what population group or work groups you are referring to							
**3. Axes of inequality**							
How are the axes of inequality addressed in each of the following objectives of the work in your service or directorate? (mark all the answers that apply)							
Objectives	Inequality axes						
	Gender	Age	Country of origin or race	Social class	Socioeconomic position (other than social class as education level)	Territory of residence	Other (indicate)
Health surveillance							
Intervention design, prioritisation and implementation							
Evaluation of interventions							
Surveillance and health control							
Final products (reports, sheet facts, letters)							
Other (indicate)							
**4. Intrasectoriality and intersectoriality**							
						Yes	No
In this service or directorate is there a need to work regularly with other areas / services of the ASPB (mark the answer)							
If YES, indicates which areas or services							
In this service or directorate, is there a need to work regularly with other areas / services EXTERNAL TO the ASPB (mark the answer)							
If YES, indicates which areas or services							
**5. Observations**							
Indicate everything you want to highlight
3. Prioritisation of organisation-wide actions was carried out following 2 methods: First, a **“**Quick and Colourful” voting session**.** This approach consisted of an open method. All ASPB staff included in the group was provided with 10 coloured stickers with which to vote. Participants gave an overview of each of the actions proposed by the world cafe workshop and were instructed to consider all the actions and to prioritise them by voting. Votes were tallied for each action and the overall scores were then rank ordered.
  Second, prioritised actions at the organisational level were assessed using a Hanlon matrix criteria [17]: 1) Magnitude, 2) Utility, 3) Feasibility, divided in the following concepts: (a) is this action opportune? (b) Human and material resources: (c) Legal regulations (d) Acceptability (e) Sustainability. The actions with the highest scores were those prioritised in their service.

4) Definition of plan objectives**:** To define the objectives of the plan, the services or departments had to include them in their annual objectives; in this way, the objectives of the plan had to be part of the objectives at their 3 levels of command (departmental, managerial and organisational).
5) Evaluation: The objectives will be evaluated at the end of 2021: 1) Performance of the prioritised actions by the departments, and 2) Satisfaction and the effect of training among participants.

Ethics approval was not required in this study.

## Results

Semi-structured interviews with 12 (60%) department heads revealed that 3 (25%) of them did not include axes of inequality or social determinants of health in their daily activities or products. The remaining 9 departments included some axes of inequality, especially those at the regional and socioeconomic level, or all axes of inequalities (age, ethnicity, socioeconomic position, gender and region).

World cafe workshops with 63 participants (37% of included workers) led to the emergence of 40 actions (Table 2) that could be grouped in 7 lines of action (Table 3).

**Table 2:** Results of the world cafe participatory workshop. Barcelona 2017–2018

Actions	
1. Improve the training of ASPB staff and clinical resources linked to social determinants, inequalities and axes of inequalities. Also include training in the perspective of gender.	
2. Provide arguments to the technical workforce to communicate the results of the process of prioritisation in the interventions when they have to interact with the general population.	
3. Improve internal training on participation techniques to adapt interventions to the needs of the population e.g. families, neighbourhoods, etc.	
4. Improve the visibility and external communication of the ASPB by providing all the necessary mechanisms such as social networks, sessions, interventions and products, and free delivery days that allow the ASPB to respond to the population’s health concerns.	
5. Systematically use easy reading and computer graphics for ASPB products and presentations in order to adapt technical discourse to the general population.	
6. Improve internal communication in the ASPB between services, programmes, circuits and ASPB resources using participatory techniques to achieve this goal.	
7. Change the ASPB services portfolio so that it better reflects what are made by the ASPB, e.g. the heath in the neighbourhoods programme, which would facilitate this program.	
8. Provide the ASPB with a checklist tool that would help to identify the inclusion of axes of inequality in ASPB interventions, programs and products.	
9. Have a person or group of people who liaise with different departments of the ASPB to support the process of incorporating the axes of inequality into the various activities of the ASPB and resolve doubts.	
10. Because of the complexity of inequalities, it was thought that a single person could not serve as a point of reference and be an expert on all the issues. This was a point to be discussed in a group of experts in the ASPB.	
11. Incorporate the gender perspective into all the activities carried out by the ASPB.	
12. Incorporate gender-based violence into the lines of work of the ASPB.	
13. Increase ASPB co-production capacity in the design and implementation of City Council policies.	
14. Participate in the decision-making process of all issues affecting the health of the city’s population by, for example, detecting cases of lead detection, infestations, participate in the decision-making tables of all those who struggle to serve environments to promote healthy leisure.	
15. Improve communication and information on the resources of the sectors of the City Council with which the ASPB regularly works, such as social services, education, housing and mobility to increase the effectiveness of joint work.	
16. Make progress in collaborative networking with districts and the key agents of the neighbourhood to allow integration of the interventions.	
17. Work to ensure that the Department of Health of the regional Government incorporates health into the school curriculum in a clear and operational manner.	
18. Promote the evaluation of the policies of the City Council to prevent gentrification and increased inequalities and generate recommendations by the sectors involved.	
19. Evaluate the impact on health as well as, for example, the impact of traffic noise on health.	
20. Prioritise ASPB interventions and/or recommendations aimed preferably at modifying the causes of health risks, rather than adapting to risk situations as is the case for example, when giving priority to actions to reduce the sources of noise instead of recommending double glazing.	
21. In assessing ASPB interventions, systematically incorporate assessment of the impact on health inequalities and that of gentrification, for example, in the case of superblocks and differences between intervening zones and adjacent zones.	
22. To work in cases of lead detection and how they generate inequalities.	
23. To think about reducing residues in our activities, interventions and programs, and not only recycling them.	
24. Include social clauses for external companies that work for ASPB.	
25. Review whether the working conditions of ASPB employees make it difficult to address social inequalities in health, and assess the measures needed to avoid or reduce them (e.g. working hours).	
26. To identify the ASPB’s working conditions to be able to carry out an organisation-wide action to improve them.	
27. Improve citizen participation in the different stages of ASPB studies or interventions by using more innovative participatory techniques that make participation easier and more attractive.	
28. Implement participatory techniques with differential approaches to ensure the co-production of certain collectives such as families, the most vulnerable groups, etc. For example, using cultural mediators to generate greater participation of vulnerable collectives.	
29. Report the results of our participatory processes to the population.	
30. Provide opportunities for internal coordination and knowledge sharing between ASPB services through innovative participatory techniques, allowing the sharing of information on actions.	
31. Adapt interventions to changing realities and make the actions more flexible so that they respond to the population’s diversity. This flexibility in interventions could improve co-production with the community as a whole. Include in the participatory diagnostics and in the design of interventions neighbourhood residents that are not organized in the work tables where technicians, organizations and neighbourhood associations normally participate.	
32. Incorporate the various axes of inequality in the interventions designed for the ASPB and those in which there is some type of participation.	
33. Propose interventions in vulnerable populations and those with which the ASPB has worked less, for example, interventions for female sex workers, since all interventions are aimed primarily at men, probably originating with the beginning of the AIDS epidemic.	
34. Increase coordination between programmes so that some interventions are shared, for example, food safety interventions could think of actions related to energy poverty.	
35. Improve collaborative relationships with other sectors or entities to share information/indicators for inclusion in the different products produced by the ASPB, e.g. indicators to make visible the problems of habitat, gentrification, etc.	
36. Improve the various information systems of the ASPB to ensure the collection of relevant data for the analysis of health inequalities.	
37. Propose collection of inequalities in the information systems of other entities, such as primary care.	
38. To define a way of systematically collecting inequitable gaps in all ASPB-dependent information sources across the organisation.	
39. Identify and improve information on vulnerable groups not only according to the region but also by defining new categories of social class or socioeconomic position. For example, people with disabilities are a precarious collective hidden within the collective of workers and very precarious workers with long working hours, low pay, and poor working conditions.	
40. Increase knowledge on the physical environment, for example, on walking, shopping, healthy eating, mobility and urbanization that allows ASPB staff to diagnose needs and design interventions.

**Table 3:** Methods and results obtained in the plan for tackling inequalities at the Barcelona Public Health Agency

Methods/participation	Questions	Results: improvement lines
Semi-structured interviews (*N* = 12 participants department heads)	For more details about the checklist see Table 1 - Are the general objectives of the service described? - Which specific population does the activity target? - Are the axes of inequality tackling? - Are intra-and intersectorality described? - What would you improve?	1. Inter- and intrasectorality at the ASPB 2. Health surveillance in services basically focused on monotoring and controlling diseases 3. Information systems 4. Knowledge transfer 5. Participatory methodologies in ASPB programmes and interventions 6. Inclusion of axes of inequality in ASPB products 7. Availability of “checklist”-type management tools to verify the incorporation of axes of inequality into ASPB products
World cafe conversation (*N* = 63 participants and 10 reporters)	- What are the determinants of inequalities in the service where you work? - What could bedone to improve our performance in relation to these determinants?	40 actions grouped in 7 lines (for more details see Table 2): 1. Define actions to improve the efforts of the ASPB to reduce health inequalities 2. Review current ASPB lines of work to detect if they include health inequalities 3. When necessary, incorporate axes of inequality into ASPB work and products 4. Increase citizen participation in ASPB action 5. Develop an internal training plan to address health inequalities 6. Develop a communication plan about health inequalities 7. Develop a cross-cutting gender plan
"Quick and colourful" voting session (*N* = 108 participants)	Multiple voting of 40 actions	31 actions with a number of votes (for more details see Table 4) 2 cross-cutting actions selected: a. Address gender-based violence from a public health perspective. b. Design and provide an internal training plan to address social inequalities
Prioritisation by services and directorates (*N* = 12 services and manegement)	Hanlon matrix: prioritization of 19 actions by services	Actions from 2019 to 2020: the 7 prioritised actions per service/managemet direction to be included in the service objectives were: 1. To make progress in collaborative networking with the districts and the neighbourhood key agents in order to integrate interventions aiming to reduce health inequalities 2. To promote assessment of the policies of the City Council to prevent gentrification and increased inequalities 3. To increase the ability of the ASPB to participate in the design, implementation and evaluation of City Council policies to reduce health inequalities 4. To incorporate axes of inequality in all the products and interventions of the ASPB 5. To improve information on vulnerable groups by defining new categories of social class or socioeconomic position 6. To include the gender perspective in all ASPB actions 7. To participate in an internal training plan to address social inequalities

*ASPB* Agència de Salut Publica de Barcelona

Priorization by “Quick and Colourful” voting allowed 108 workers (63%) in the selected areas of the ASPB to participate in the session resulting in 31 actions being ordered by votes (highlight actions with more than 30 votes can be seen in Table 4). The 31 voted actions were grouped in 19 actions by 2 professionals of the leading group. These 19 actions were prioritised by the services and directorates using the modified Hanlon matrix and the following 7 actions were prioritised (Table 3):

1. To make progress in collaborative networking with the districts and the neighbourhood key agents in order to integrate the interventions aiming to reduce health inequalities.
2. To promote evaluation of the policies of the City Council to prevent gentrification and increased inequalities.
3. To enhance the ability of the ASPB to participate in the design, implementation and evaluation of City Council policies to reduce health inequalities.
4. To incorporate the axes of inequalities in all the products and interventions of the ASPB.
5. To improve information on vulnerable groups by defining new categories of social class or socioeconomic position.
6. To incorporate the gender perspective in all ASPB actions.
7. To participate in an internal training plan to address social inequalities.

**Table 4:** Results of the “Quick and Colorful” voting prioritization session of actions to improve tackling health inequalities in the Barcelona Public Health Agency, Barcelona 2017–2018

Lines	Actions	Number of votes	Carried out by
Incorporate axes of inequality into the ASPB work and products.	Design a checklist that allows you to review ASPB products and actions.	22	Services
	**Incorporate axes of inequality in all the products and interventions of the ASPB.**	**33**	**Services**
	Improve the various information systems of the ASPB to ensure analysis of health inequalities.	20	Services
	Improve the information on vulnerable groups by defining new categories of social class or socioeconomic position.	26	Services
	Improve the information and collection systems that do not depend on the ASPB.	20	External
	**Increase coordination between programmers so that some interventions are shared as follows.**	**62**	**Services**
Increase citizen participation in ASPB action	Improve citizen participation by using more innovative participatory techniques.	30	Services
	**Guarantee participation of the most vulnerable collectives with participatory techniques with differential approaches.**	**59**	**Services**
	**Report the results of our participatory processes to the population.**	**32**	**Services**
Define actions to improve the work of the ASPB	Define an intersectional management strategy.	5	Services
	Improve information on the resources of the sectors of the City Council with which the ASPB works regularly.	11	Services
	**Make progress in collaborative networking with the districts and the neighbourhood key agents in order to integrate interventions.**	**40**	**Services**
	**Increase the ability of the ASPB to participate in the design, implementation and evaluation of City Council policies.**	**46**	**Services**
	**Promote the evaluation of the policies of the City Council to prevent gentrification and increased inequalities.**	**37**	**Services**
	**Work to promote the incorporation of health in the school curriculum in a clear and operative way by the Department of Health.**	**90**	**External**
Perform an internal training plan	**Design an internal training plan to address social inequalities.**	**33**	Organisation-wide
	Define an internal training plan from the gender perspective.	19	Organisation-wide
	Define an internal training plan in participatory techniques	13	Organisation-wide
	Define an internal training plan for intersectional action.	11	Organisation-wide
	Organize a cross-cutting group with consultative functions.	4	Services
Develop a cross-cutting gender plan	Gender equality plan.	6	Services
	**Gender perspective on all ASPB actions.**	**44**	**Services**
	**Address gender-based violence from a public health perspective.**	**65**	Organisation-wide
Develop a communication plan	Define an ASPB communication plan.	6	Communication area
	**Improve the visibility and external communication of the ASPB.**	**37**	**Communication area**
	**Systematically use tools such as easy reading to adapt technical discourse to the general population.**	**52**	**Communication area**
	**Improve organization-wide communication within the ASPB using participatory techniques.**	**62**	**Communication area**
Review current ASPB lines of work	Identify areas of work in which action could be taken to improve the approach to inequalities in ASPB services.	23	Services
	Review the portfolio of services of the ASPB to better respond to the reality of social inequalities in health.	30	Services
	**Identify working conditions in the ASPB.**	**36**	**Human resources manager directorate**
	**Assess the measures needed to avoid or minimize inequalities in the workplace (e.g., working hours).**	**101**	**Human resources manager directorate**

Note: Actions with more than 30 votes are highlighted in bold

These actions were included in the annual objectives of the 12 departments and services to be achieved in 2021. The methods and results are summarised in Table 3.

### Actions already implemented

1. *Internal training plan to address social inequalities*

During the third quarter of 2018, 2019 and until March 2020 (before the covid-19 pandemic), a basic training module on social determinants of health and health inequalities was designed, consisting of a theoretical part and another part adapted to the needs of staff attending the training module. At the time of this publication, 5 training workshops have been held, attended by 100 people (30% of staff). At the end of 2021, 160 workers (48% of staff) had been trained. Because we have been recording satisfaction and pre- and post-training data, we will be able to evaluate the effect of training on participants’ knowledge of social inequalities.

As a consequence of the training plan, for 2020, the Departments of Environmental Quality and Intervention and Health Information Systems decided to review the existing scientific evidence on environmental health inequalities, to review the availability and current use of indicators at the ASPB, and finally to define a road map to improve information on inequalities in environmental health.

2) *Addressing gender-based violence from a public health perspective*

At the beginning of 2019, a working group on gender violence was organized at the ASPB aiming to: review the existing evidence to develop a conceptual framework [18] and to analyze gender-based violence in the context of couples in the city of Barcelona.

3) *Annual planning*

Targets related to the Plan were included in the annual planning of all services and directorates in 2019, 2020 and 2021.

## Discussion

The results show how a local public health organisation can successfully introduce health inequalities in the actions of diverse areas of the organisation through participatory methods. The diagnosis showed that health inequalities were unequally addressed and not always with the same intensity. Therefore, tasks were prioritized to level up the activities implemented in all departments and mainly in those not yet implementing them. This study confirms that the approach to inequalities in health must be carried out at both the organizational level and at the level of specific public health departments.

In Barcelona, policies for the reduction of health inequalities have been undertaken in the last years, as in other cities [19], although bureaucratic restraints and resistance from various levels of the administration have been described as important barriers [9]. In addition, verticality in decision making has negative consequences for compliance with reducing inequalities [13]. Our plan is a good example of how an organisation can not only address health inequalities in different public health areas, but also of how improvement actions can be achieved from the participatory process. In this regard, in our review of the scientific literature, we found no similar cases to that described herein.

In our experience in the city of Barcelona, it is very difficult to align all the stakeholders in tackling health inequalities [2] e.g. in the ASPB some departments are more ahead of others in describing inequalities in health [20].

In our participatory plan, the public health workforce assessed health inequalities as substantial and expressed the need to incorporate health determinants and health inequalities into the activities and products of the ASPB. Public health information systems collect little information about social class and other axes of inequality [21]. In addition, it is important to have data from information sources which are not common, e.g. data from environmental contexts [22].

Another improvement action was to increase citizen participation in ASPB action. An emerging body of research suggests that actively engaging affected stakeholders has the potential to make a positive difference in achieving outcomes [23]. One of the keys to enhancing citizen participation is providing citizens with access to better information that is easily understood [24].

Intersectoriality is expressed by workers as the need to make progress in collaborative networking with the districts and the neighbourhoods and also to increase the ability of the ASPB to participate in the design, implementation and evaluation of City Council policies. Some of the problems identified with intersectoral working are the persistence of the traditional perspective and the lack of multi-intersectoral knowledge, co-operation and function between sectors and stakeholders. However, members of the ASPB believe that intersectorality would have a positive effect on health inequalities [17]. To be effective, the processes of collaborative governance underlying area-based programmers require the attention of the local authority, including the creation and governing of networks, a competent public health workforce and supportive infrastructures [13].

### Training in health inequalities

The participatory process revealed that not all the workforce in the ASPB had the same background and knowledge for dealing with health inequalities. The participatory methods also revealed the need to develop an internal training plan in health inequalities. Currently, due to the plan, some relevant training is already taking place, for example in basic concepts of social determinants of health, axes of inequality, and health inequalities, etc. Much of this education tends to focus on a specific department [12]. There is evidence that training health professionals about social determinants of health generates awareness of the potential root causes of health inequalities and the importance of addressing them both in and with communities [25]. There is also evidence that public health professionals must develop appropriate skills and attitudes to be advocates for change [26].

To implement this plan, the ASPB will need to devote both human and economic resources in both the medium and long term to achieve its objectives. In addition, ASPB workers, as well as the various departments, will be in different phases of the execution of the plan: While some workers have already received training and can contribute to the improvement actions of their department, others have yet to receive training.

### The gender perspective

The public health workforce in the city of Barcelona and in other high-income countries is predominantly female. One of the most voted actions was related to avoiding or minimizing inequalities in the workplace, showing the need for women with children in their care to balance work and personal life [27]. Another policy for gender equity that ASPB has taken into account is gender responsive budgeting to best respond to the actions prioritised by its workforce. Europe provides many examples [22, 28] to be followed by the ASPB. In addition, the Barcelona City Council has approved the gender equality plan for the city [29].

ASPB workers assign high priority to the prevention of gender-based violence from the public health perspective. Policies to reduce health inequalities are rarely included to reduce gender violence in strategic actions [30]. In addition, the inclusion of the gender perspective in actions of the ASPB was highly prioritised by its workers. Last year’s gender perspective has therefore been introduced by City Council and into the policies currently being implemented in the city.

### Learned lesson of the COVID-19 pandemic

The participatory process of the plan’s implementation has been finished before COVID-19 pandemic. However, there are some lessons learned in the last 2 years of the pandemic due to the links of it with inequality in health. The COVID-19 pandemic, described as a syndemic, allows us to rethink the relationship between human health and planetary health. The pandemic affects populations worldwide but, although everyone is susceptible to the virus, there are numerous evidences of having a greater impact on lower socioeconomic groups and minorities. As we state in this plan, it is essential for public health administrations understanding inequalities to develop policies to tackle them. A conceptual framework helped us to identify and explain the interactions between biological, social, and environmental factors as part of the structural and intermediate determinants involved in this pandemic [31].

Another lesson learned that derived from the pandemics is that the classic model of epidemiological surveillance has been overwhelmed by the pandemic. A new approach requires not only the guidance of the experts in virology or epidemiologists but also that of other professionals in public health and other disciplines with a broader vision of the connection between political, social and economic factors and infectious diseases [32]. In addition, during the pandemic, the indicators produced by the health administration of Barcelona clearly showed the unequal distribution of incidence of COVID-19 and the policy responses to them [33].

### Limitations

One of the limitations of this plan, and specifically of participatory methods, is that the participants’ own social status or limited or insufficient information may result in a lack of variability. However, participatory methods see people as active agents and include dimensions previously not applied in other approaches. In addition, participatory methods were conducted with the participation of a wide range of the workforce.

Another limitation is that the results could reflect the existing power relations in the community. In our case, department heads and people with decision-making responsibility were excluded from the participatory sessions with the workers, leaving the session exclusively for them.

Some areas of the ASPB, such as health protection and laboratory analysis services, did not participate in the process. However, there have been multiple reflections and interpretations on food safety from the point of view of equity that need to be included in the health protection departments of the ASPB in the near future as part of this plan.

## Conclusions

We conclude that to reduce social inequalities in health, the evidence indicates that political commitment is essential. In addition, the participation of ASPB public health professionals produced a plan of improvement actions to address inequalities in a participatory manner and almost certainly with a greater capacity to involve ASPB workers than if these measures had been proposed by the ASPB management. In the future, the progress of this plan will need to be assessed, both in terms of process and results. The experience shown in this article could be useful for other municipalities that have included tackling inequalities in health in their political agenda.

## Data Availability

The datasets used and/or analysed during the current study are available from the corresponding author on reasonable request.
